# Rapid and Reliable Quantification of Prime Editing Targeting Within the Porcine *ABCA4* Gene Using a BRET-Based Sensor

**DOI:** 10.1089/nat.2022.0037

**Published:** 2023-06-02

**Authors:** Tobias Wimmer, Hannah Sawinski, Anne M. Urban, Jan Motlik, Knut Stieger

**Affiliations:** ^1^ Department of Ophthalmology, Justus-Liebig-University Giessen, Giessen, Germany.; ^2^ Institute of Animal Physiology and Genetics, Academy of Sciences of the Czech Republic, Libechov, Czech Republic.

**Keywords:** *ABCA4*, genome editing, prime editing, cellular reporter assay, bioluminescence resonance energy transfer

## Abstract

Stargardt disease (STGD) leads to blindness in children and young adults. So far, no curative therapy is available and gene augmentation therapies have not yet advanced to the clinics, in part, due to the limited packaging capacity of adeno-associated viruses used to transfer genes into photoreceptor cells. Prime editing offers a new perspective to treat mutations on the genomic level. A nicking variant of Cas9 fused to a reverse transcriptase complex with an elongated guideRNA force intracellular mismatch repair to correct the targeted mutation even in postmitotic cells such as photoreceptors in the eye. Using a custom-made bioluminescence resonance energy transfer (BRET)-based editing sensor in HEK293 cells, we tested 27 different prime editing guide RNAs (pegRNAs) and additional 4 nicking guide RNAs (ngRNAs) with regard to their efficiency to induce sequences changes in exon 43 of the porcine ATP binding cassette subfamily A member 4 (*ABCA4*) gene that eliminate a mutagenic adenine frameshift insertion, which has been associated with STGD in humans. We identified nine working pegRNAs, and in combination with ngRNAs, we achieved a correction rate of up to ≈92% measured with the BRET-based reporter system. Our data prove the high efficiency of prime editors to correct mutations and highlight the importance of optimal ngRNA design, thus offering a promising editing tool to correct *ABCA4* mutations in the disease context.

## Introduction

Stargardt disease (STGD), an inherited autosomal recessive retinal degeneration, is the most common cause of vision impairment and blindness in children and young adults with a prevalence of 1:8,000–10,000 newborn. Mutations in the ATP binding cassette subfamily A member 4 (*ABCA4*) gene are associated with STGD (OMIM: Entry—#248200—STARGARDT DISEASE 1) leading to bilateral loss of central vision, a delay in dark adaption, and an atrophy of the macula [1,2]. The ABCA4 protein is located at the rim of the discs and invaginations in the outer segments of photoreceptors and is involved in the visual cycle. Nonfunctional ABCA4 leads to the accumulation of all-trans- and 11-cis-retinoids in the photoreceptors, and bis-retinoids in the retinal pigment epithelium (RPE) leading to RPE and the subsequent photoreceptor degeneration [2,3].

The *ABCA4* gene contains 50 exons and is spanning ∼128,000 bp on chromosome 1, and its full coding transcript size contains 7,328 bp. The *ABCA4* gene is highly conserved among species; the human *ABCA4* gene (NP_000341.2) shares an 86.19% homology to the porcine *ABCA4* gene (XP_020945843.1). To date, no curative therapy is available for *ABCA4* loss-of-function mutations.

Prime editors and base editors, which consist of a nicking mutant of SpnCas9 (*Staphylococcus pyogenes* Cas9 H840A) fused to an optimized reverse transcriptase or deaminases, respectively, have recently revolutionized the field of genome editing [4]. Nicking (single-strand break) of a double-stranded DNA does not lead to high mutagenic indel formation as seen at repaired DNA double-strand break (DSB) sites, thus allowing the comparably safe approach to correct point mutations or small insertions/deletions [5].

The prime editor system (PE2) uses an extended RNA [prime editing guide RNA (pegRNA)], which is not only responsible for guiding the Cas9/gRNA complex to the genomic target sequence, it also serves for binding to a 3′Flap after nicking (prime binding site) and also as a template [reverse transcriptase template (RTT)], which consist of the desired edit, for the reverse transcriptase. This results in an equilibrium of the edited 3′-Flap and unedited 5′-Flap, leading to a heteroduplex after repair that determines the editing outcome. To further increase editing, nicking of the opposite, nonedited strand with an additional nicking guide RNA (ngRNA) can enhance installing the edit (PE3 and PE3b systems). Using the combination of nicking at different positions and strands significantly reduces unwanted indel formation due to mutagenic DSB repair [4].

High editing efficiencies are crucial for any relevant treatment approach to enter the clinical stage. Measuring these efficiencies is often difficult to achieve in a robust manner. Fluorescent or luminescent reporter systems are not commonly used in genome editing research, but they are of help in obtaining rapid and reliable access to data of endonuclease-mediated correction of disease causing mutations. These reporter systems are usually based on a reading frame disruption or restoration. In early developmental stages, these reporter systems can provide useful data without the need of a mutated cell line. Especially when specific cell lines such as photoreceptor cell cultures are missing, episomal reporter systems that function in standard cell lines can be used to verify the desired outcome instead of using next-generation sequencing or tracking of indels by decomposition (TIDE)/Interference of CRISPR Edits/Indel Detection by Amplicon Analysis.

Nevertheless, the information content of these reporters is limited and does not replace the mentioned techniques in *in vivo* experiments [6–8].

We have developed a bioluminescence resonance energy transfer (BRET)-based reporter system that allows robust and fast assessment of activity of any form of guide RNA and editing system at the target site [8]. BRET allows precise measurements in a cellular context after transfection and offers some advantages over fluorescent reporter assays used in previous genome editing studies. BRET uses simultaneous measurements of both BRET components RLuc8 and GFP2, where the RLuc8 light output serves as an internal standard normalizing the variable GFP2 output [9]. Furthermore, the ratiometric BRET ratio eliminates concentration and transfection fluctuations making this system ideal for *in vitro* experiments [10–12].

Applying genome-editing approaches to correct disease-causing mutations needs meaningful model systems when translating *in vitro* results to *in vivo* experiments. Mouse models are very important in basic research when trying to understand disease mechanisms, but are limited in mimicking human disorders. Pig models in contrast are ideal to study human diseases, because their physiology and anatomy are comparable with humans [13]. Especially in retinal research, the size of the eye, specific anatomic features of the retina, and the immune status have great influence in optimizing the therapeutic procedure. In addition, the pig genome shows a higher similarity to the human genome compared with the mouse genome [14].

In this study, we describe the editing quantification of a porcine *ABCA4* frameshifting adenine insertion using prime editors 2, 3, and 3b using our custom-made BRET-based reporter system and highlight mechanistical impacts on the system with regard to direct locations of involved ngRNAs.

## Materials and Methods

### Target sequence synthesis

Porcine wild-type target DNA containing terminal restriction sites (*Avr*II/*Bsi*WI) for cloning into the BRET reporter system was synthesized according to a modified GeneBuilder (Thermo Fisher, Dreieich, Germany) protocol. In brief, overlapping oligonucleotides were designed (https://hpcwebapps.cit.nih.gov/dnaworks/), hybridized, and polymerase chain reaction (PCR) amplified using flanking 5′ oligonucleotides as primers (Supplementary Table S2.1 and Supplementary Fig. S1). The resulting sequence was subcloned into pTOPO (Invitrogen, Karlsruhe, Germany), restriction digested (*Avr*II/*Bsi*WI), and inserted into the BRET reporter plasmid. A frameshift mutation was installed by the insertion of an adenine (GTG→GTAG) by mutagenic PCR (Phusion polymerase; Thermo Fisher Scientific, Darmstadt, Germany) using counter-directed primers, one containing the adenine at the 5′ end (primer and oligonucleotides: Supplementary Table S2.2).

### pegRNA and ngRNA design and cloning

The porcine *ABCA4* exon 43 DNA sequence as a human homologous prime editing target was obtained from breeding animals used in animal model generation after DNA extraction, PCR amplification with exon-specific PCR primers, and verification by Sanger sequencing. The sequence containing the frameshifting adenine insertion was used to design possible pegRNAs and ngRNAs using PrimeDesign (Supplementary Table 1.1 and 1.2) [15]. PegRNA cloning was carried out as previously described [4]. In brief, complementary oligonucleotides for Spacer-, Scaffold-, and 3′-Extension-Sequences were hybridized and cloned into the *Bsa*I-HFv2 linearized pU6-pegRNA-GG-Acceptor plasmid via Golden Gate cloning. Complementary ngRNA oligonucleotides were hybridized and cloned into a U6-promotor containing plasmid via *Bbs*I restriction.

### Cell culture, transfection, and cell lysis

HEK293-T cells (*Homo sapiens*; ATCC: CRL-3216) were obtained from the American Type Culture Collection. Cells were maintained in Dulbecco's modified Eagle's medium (DMEM), supplemented with l-glutamine (4 mM; PAN Biotech, Aidenbach, Germany), pen/strep (100 IU/mL/0.1 mg/mL; Anprotec, Bruckberg, Germany), and 10% fetal bovine serum (PAN Biotech) in a humidified incubator at 37°C and 5% CO_2_. At confluence, cells were split in 1:10 ratio by detaching the cells with Accutase (Anprotec, Bruckberg, Germany).

Transient PEI transfection (polyethylenimine; MW: 25 kDa; Polyscience, Hirschberg, Germany) was used in all experiments in this study. HEK293-T cells were seeded at a density of 200,000 cells per well in 6-well plates (Greiner, Frickenhausen, Germany) the day before transfection. On the day of transfection, culture media were replaced with 1.5 mL of fresh and supplemented DMEM. Plasmid DNAs (pBRET reporter: 500 ng; pCMV-PE2: 750 ng; pegRNA plasmid: 250 ng; ngRNA: 83 ng) were pipetted to 100 μL of sterile 150 mM NaCl followed by the addition of 200 μL of 1.0 mg/mL PEI. After an incubation of 10 min at room temperature (RT), the transfection mix was added dropwise to the cells. Six hours post-transfection, the culture media were replaced with 2 mL of fresh one and the cells were incubated for additional 24–72 h at 37°C in a humidified incubator.

For cell lysis, culture media of transfected cells were discarded and cells were washed once with sterile, ice-cold 1 × phosphate-buffered saline (PBS). Cells were collected using a cell scraper after 100 μL of luciferase assay lysis buffer (Promega, Mannheim, Germany) was added per well. Cells were transferred into a fresh tube and lysed in liquid nitrogen in two freeze–thaw cycles. Samples were cleared at 17,000 *g*, at 4°C for 10 min, and a supernatant was used for further experiments.

### Western blot

Western blot was performed to verify the expression of prime editors in HEK293-T cells with an antibody raised against SpCas9 (mouse anti-CRISPR-Cas9; ab191468; Abcam, Cambridge, United Kingdom). HEK293-T cells transfected with PEs were lysed as described above. Samples containing 20 μg of whole protein were separated in a 10% SDS-PAGE (sodium dodecyl sulfate–polyacrylamide gel electrophoresis) at 150 V for 75 min and transferred via semidry blotting onto nitrocellulose at 75 A for 60 min. Blocking (5% milk powder in 1 × PBS) was carried out for 1 h at RT, followed by an incubation with anti-SpCas9 (dilution: 1:1,000 in blocking buffer) overnight at 4°C and an incubation with a secondary horseradish peroxidase (HRP)-labeled anti-mouse IgG (Sigma-Aldrich, Taufkirchen, Germany) at RT for 90 min. After the addition of the HRP substrate (Amersham Bioscience, Taufkirchen, Germany) the signal was detected for 10 min on a chemiluminescence film (Amersham Bioscience, Taufkirchen, Germany).

Western blot-based verification of the BRET reporter frame correction was carried out as described above, but with a rabbit anti-RLuc antibody (Biomol, Hamburg, Germany) followed by an incubation with the anti-rabbit IgG-HRP (Sigma-Aldrich).

### Fluorescence microscopy

PE transgene- and BRET reporter GFP2 expression was verified with fluorescence microscopy (Keyence BioZero, Neu-Isenburg, Germany) with constant exposure times of 0.8 s.

### BRET assay

The cellular BRET assay was performed after cell lysis with equal sample volumes. In brief, 20 μL of cell lysates was plated onto white 96-well plates (COSTAR Lumiplates FlatWhite; Corning, Kaiserslautern, Germany). Measurements were performed using the Tecan Infinite M1000Pro platereader (Tecan, Groeding, Austria) after automated injection of 100 μL of renilla luciferase substrate (5.0 μg/mL coelenterazine 400a; NanoLight, Inc., Pinetop) per well in the dual-luminescence mode with two separate transmission filters (blue filter: 370–450 nm; green filter 510–540 nm).

### TIDE analysis of editing events

To verify the obtained BRET reporter results, sequence-based TIDE was used to additionally quantify −1 deletions, which refers to editing efficiency. Remaining plasmid DNA was isolated from transfected cells using a plasmid kit (M&N, Dueren, Germany). BRET reporter target fragments of ∼600 bp were amplified using PCR with primers located in the RLuc8 (5′-attgtccgcaactacaacgcctac-3′) and the GFP2 (5′-tcggggcggatgtacacgttg-3′) reading frame. PCR products were sent to a commercial laboratory for Sanger sequencing (Seqlab, Goettingen, Germany) and analyzed with the TIDE online analysis tool (http://shinyapps.datacurators.nl/tide/).

### Statistics and calculations

All date presented are shown as mean ± standard deviation and represent *n* = 3 biologically independent samples with quadrupole measurements, unless otherwise stated. Direct comparisons were analyzed statistically with *t*-test. Multiple comparisons were performed using ANOVA. *P* values <0.05 were considered statistically significant, and *P* values <0.001 as highly significant. Statistics are calculated with SigmaPlot (Systat Software, Erkrath, Germany).

The BRET ratios were calculated using the measured intensities I_[GFP2]_/I_[RLuc8]_. Wild-type BRET reporter served as 100% editing control, while the BRET reporter harboring the frameshift mutation served as control for normalization (0% editing).

## Results

**FIG. 1.**
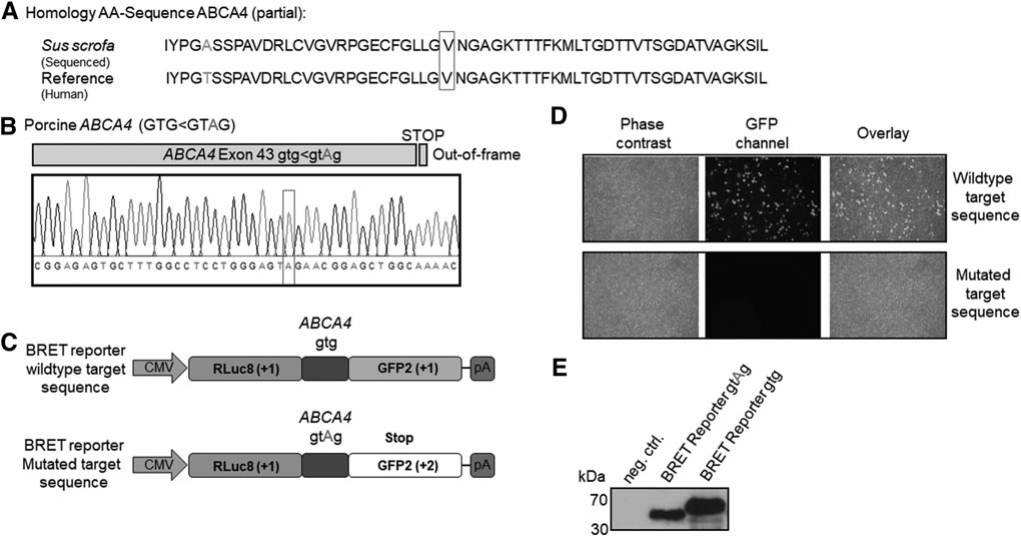
Prime edit target sequence integration into BRET reporter system and characterization. **(A)** Homologous porcine (*Sus scrofa*) (XP_020945843.1) and human (XP_047272660.1) ABCA4 amino acid target sequence coded by exon 43. **(B)** Sequenced (gtg<gtAg) frameshifting adenine insertion in porcine *ABCA4* exon 43. **(C)** Integration of wild-type and mutated sequence into the BRET reporter system. **(D)** Frameshift confirmation of the integrated target sequence by fluorescence microscopy after transient transfection in HEK293-T cells. **(E)** Frameshift caused premature stop codon reducing molecular weight of the BRET reporter after transient transfection in HEK293-T cells, cell lysis, and the detection with an anti-RLuc8 antibody after western blot. ABCA4, ATP binding cassette subfamily A member 4; BRET, bioluminescence resonance energy transfer.

Our target in this study is a frameshift mutation in the porcine *ABCA4* gene at position 1973. The amino acid sequence in this area of the gene is well conserved among different species including humans (Fig. 1A) and encodes for parts of the NBD2 domain. The analyzed sequence represents exon 43 on the genomic level. An adenine insertion (gtg<gt**A**g) causes the frameshift leading to a premature stop codon (Fig. 1B).

To apply the BRET reporter system for quantification of editing events at the target site, the wild-type *ABCA4* exon 43–44 sequence was synthesized (oligonucleotides for synthesis, Supplementary Table S2.1) and cloned in-frame in our previously described BRET reporter system between the RLuc8 and the GFP2 reading frames [8]. The target frameshift mutation has been installed with mutagenesis PCR (PCR primer, Supplementary Table S2.2) leading to a frameshift of the BRET acceptor gene GFP2 (Fig. 1C). We analyzed correct wild-type in-frame cloning and the presence of adenine insertion leading to the frameshift in the GFP2 gene by fluorescence microscopy after transfection of HEK293-T cells. Green fluorescence showed the correct insertion of the wild-type target sequence, while the absence of green fluorescence was observed after mutagenesis indicating a disturbed GFP2 expression (Fig. 1D).

To verify the expression of both reporter variants, we performed a western blot showing a reduced molecular weight (calculated MW: 37.4 kDa) after adenine insertion in comparison with the wild-type sample (calculated MW: 64.1 kDa) proving the presence of the premature stop codon due to the frameshift (Fig. 1E).

**FIG. 2.**
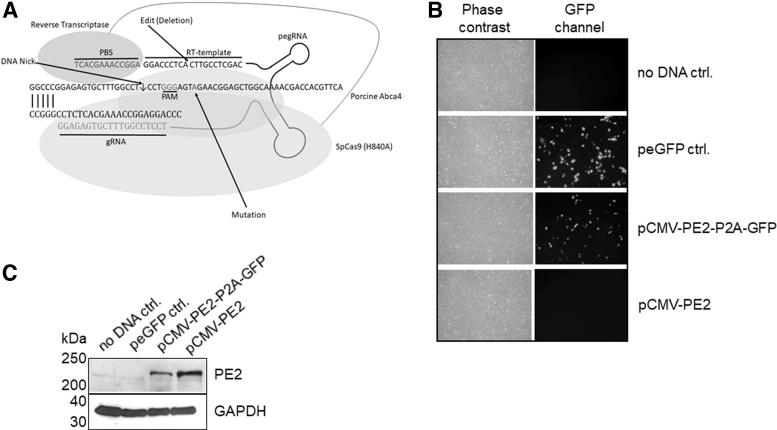
Prime editor expression. **(A)** Prime editor as a fusion of nicking SpCas9 (H840A) with a reverse transcriptase, complexed with the edit containing pegRNA bound to porcine ABCA4 target DNA. **(B)** Fluorescence microscopy after transient HEK293 transfection with PE2 expressing plasmids. **(C)** Western blot-based verification of PE2 expression with an anti-SpCas9 antibody. pegRNA, prime editing guide RNA.

For the verification of PE2 (Fig. 2A) expression in HEK293-T cells, we transfected the cells with plasmids coding for PE2 (pCMV-PE2) alone and a GFP containing PE2 (pCMV-PE2-P2A-GFP) separated by P2E (self-splicing element) and analyzed expression with fluorescence microscopy and western blotting. For pCMV-PE2-P2E-GFP, we could show green fluorescence indicating GFP and PE2 expression. For pCMV-PE2, no fluorescence was measured (Fig. 2B). Western blotting using an anti-SpCas9 antibody showed immunoreactive material at the calculated size of ∼220 kDa for both pCMV-PE2 and pCMV-PE2-P2A-GFP, indicating expression in our cell culture model HEK293-T (Fig. 2C).

Overall, we designed and cloned 27 pegRNAs with different combinations in gRNA sequences, RTT- and PBS-length for the PE2 system, as well as 4 additional ngRNAs for the PE3 and PE3b system (Supplementary Table S1.1 and S1.2).

**FIG. 3.**
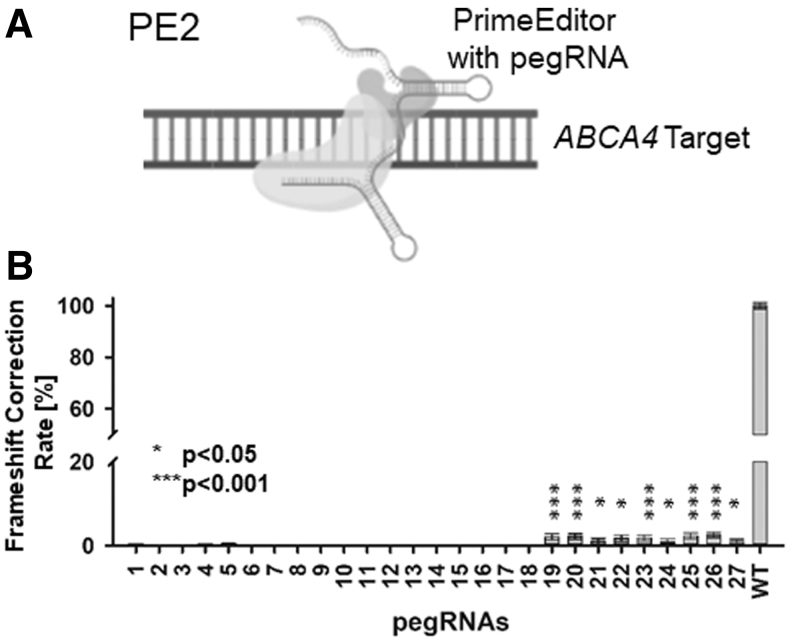
Prime editor 2. **(A)** Schematic representation of PE2 on the mutated target sequence. **(B)** Frameshift correction rate [%] of PE2 complexed with 27 different pegRNAs compared with the wild-type target sequence containing BRET reporter as 100% control (*P < 0.05; ***P < 0.001).

Next, we applied PE2 in combination with our 27 pegRNAs (Fig. 3A) to our target BRET reporter to measure editing of the previously manipulated, mutagenic porcine *ABCA4* target sequence to obtain the editing rate [%]. For pegRNAs 1–18, no significant editing rate was observed compared with our controls, while significant (*P* < 0.05 and *P* < 0.001) editing rates were achieved for pegRNAs 19–27 (Fig. 3B and Supplementary Fig. S2A, B).

**FIG. 4.**
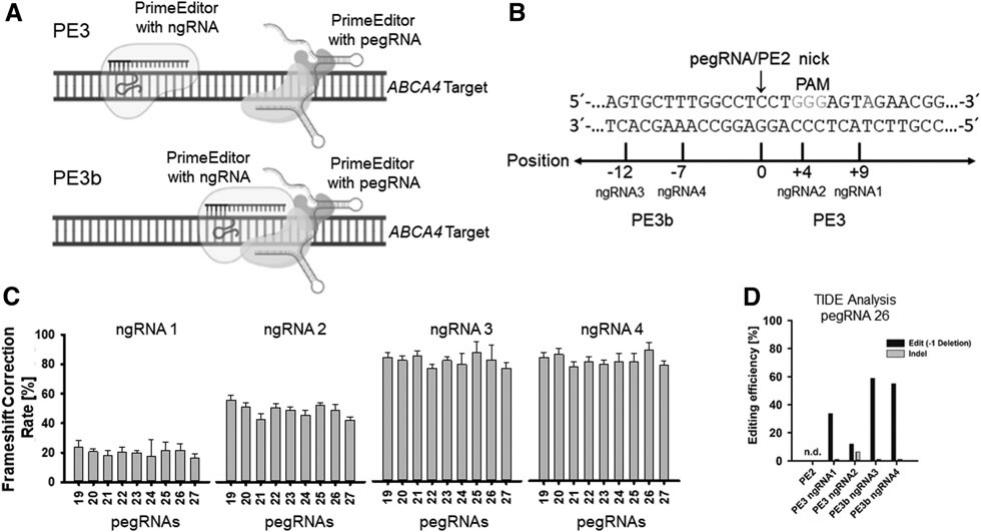
Prime editors 3 and 3b. **(A)** Schematic representation of PE3 and PE3b on target sequence with additional nicking outside (PE3) and inside (PE3b) the target seed. **(B)** Positions of additional nicking gRNAs of PE3 and PE3b. **(C)** Frameshift correction rate [%] of selected pegRNAs (19–27) with two PE3- and two PE3b-ngRNAs. **(D)** TIDE analysis of prime editor-mediated editing (−1 deletion) with combined indel analysis. ngRNA, nicking guide RNA; TIDE, tracking of indels by decomposition.

Nicking the nontargeted strand with additional ngRNAs (PE3, PE3b) directs DNA repair using the edited strand as a template (Fig. 4A) [4,15]. To probe whether this approach is able to increase the editing rate, we transfected four additional ngRNAs targeting different positions in relation to the initial pegRNA-mediated nick (Fig. 4B) with the PE2 system and pegRNAs 19–27 and analyzed editing by fluorescence microscopy and the BRET assay again. Interestingly, we already observed a massive increase in fluorescent cells after cotransfection for each ngRNA tested and compared with the PE2 control (Supplementary Fig. S3). The BRET assay confirmed our observation in fluorescence microscopy with an increased editing rate of up to 91.2% for pegRNA26 in combination with ngRNA4 (Fig. 4C).

Additional nicking with the PE3 and PE3b system improved the editing rate massively compared with the PE2 system for all of our four ngRNAs tested in a position-specific manner. While ngRNA1 and ngRNA2 are nicking the DNA substrate outside the guideRNA seed sequence, ngRNA3 and ngRNA4 are additionally nicking the gRNA seed sequence. Nicking outside the seed improved editing rate to >60% for ngRNA1 and >30% for ngRNA2, respectively. Using the PE3b system with seed nicking further increased editing to >80% (Fig. 4C). Nicking the nonedited DNA strand overall increased the editing efficiency significantly (*P* < 0.001) with all ngRNAs tested (Supplementary Fig. S4). Interestingly, the position of the additional nick influences the editing efficiency. When nicking the seed, a shorter distance to the pegRNA nick seems to be beneficial, as shown with ngRNA3 and ngRNA4 with an upstream distance of −12 and −7 nucleotides.

In contrast, nicking outside the seed generates an opposite effect. Nicking at a longer distance from the pegRNA nick with ngRNA1 and ngRNA2 decreases (downstream +4 and +9 nucleotides) efficiency.

Finally, the efficiency rate of the PE3-pegRNA26/ngRNA4 combination was verified at the protein level with a western blot (Supplementary Fig. S5) and at the DNA level by TIDE analysis. Successful editing corrects the BRET reporter reading frame, shifting the immunoreactive band to a higher molecular weight. Consequently, we observed a great portion of the corrected BRET reporter at the expected molecular weight (Supplementary Fig. S5). TIDE analysis revealed no detectable editing with PE2, while the BRET reporter assay detected editing, although at a minor rate. In contrast, for PE3/PE3b systems, the editing efficiencies quantified by TIDE ranged at around 60% (Fig. 4D). The values are generally lower compared with the data obtained with the BRET reporter, but display a similar pattern, where 3b prime editors using ngRNA3 and ngRNA4 showed the highest modification rates.

In addition, TIDE also allowed an insight into indel distribution. Only minor indels ranging from 1.1% to 6.7% could be detected in PE3/PE3b samples (Fig. 4D).

## Discussion

In this study, we report the editing efficiencies of prime editing approaches at the porcine *ABCA4* locus addressing a frameshift mutation using a quantitative BRET reporter system. By use of our custom-made BRET-based system, we were able to quantify the efficiencies of 27 pegRNAs in combination with 4 different ngRNAs and identified 4 lead targets for further testing in the subsequent *in vivo* models.

Almost no editing was observed when using the PE2 system in 18 out of 27 pegRNAs tested. This indicates absence of installation of the desired edit located in the RTT possibly due to a favoring of the nonedited strand for single-strand DNA repair. In contrast, peg RNAs 19–27 showed partially a highly significant increase in editing (Fig. 3B). Interestingly, the gRNA and the PBS portion of the pegRNA do not seem to influence the outcome. However, the longer RTT portions seem to improve PE2-mediated editing significantly (Supplementary Table S1.1).

The PE3 and PE3b systems use additional nicking with an ngRNA outside or inside the target seed on the opposite nonedited strand. This leads to a shift of the single-strand DNA repair using the edited strand to install the edit with different efficiencies. In this study, only the position of the additional nicking influences the editing efficiency. Nicking with the PE3 system already supported installing the edit significantly, but nicking within the target seed leads to superior editing efficiencies in all pegRNA/ngRNA combinations tested. Even the PE3b nicking position within the target seed seems to alter the outcome, and additional nicking in close proximity of the initial pegRNA-mediated nick slightly increased editing.

Double nicking in close proximity raises the question whether a full DSB is generated in the target sequence, especially when using the PE3b system. This would result in the activation of DSB repair mechanisms, such as error-prone nonhomologous end-joining at the target site. However, a recent study revealed that among all the different DNA repair pathways present in the cell, only mismatch repair is crucial in prime editing-meditated DNA nicking [16,17]. Consequently, prime editing offers an elegant way to correct mutations with reduced indel formation and off-target activity, compared with Cas9 approaches where an induced DSB is repaired with different competing repair pathways rendering the desired outcome in dependence of cell cycle status and the presence of repair templates uncertain [4,18].

A disadvantage of the prime editing system is the length of the coding sequence containing the nCas9 fused to the reverse transcriptase with its regulatory sequences together with the cassettes coding for the pegRNA and the ngRNA. This cDNA does not fit into a single adeno-associated virus (AAV) with a capacity of ∼4.7 kbp, which is generally used to transduce postmitotic photoreceptors in the eye. Dual AAV systems using trans-splicing or homologous sequences are able to overcome this issue and are currently under development by several groups for this application.

Reporter systems are not widely used in genome editing research, but especially the BRET-based reporter system generated for this study can provide valuable information about the different outcomes generated by the different prime editors, without the need of a mutated cellular model [8]. Certainly, fluorescence-based reporter systems such as the BRET reporter used in this study are generally less powerful tools compared with sequencing-based analysis methods such as TIDE or NGS. Especially, in the case when using the PE3/3b system with a reported indel rate of up to 10%, BRET reporters cannot differentiate between the desired adenine deletion and potential indel generation after a double nicking DSB event was generated and coincidentally corrected to re-establish the reading frame.

However, the robustness of the assay, the rapid application, and the possibility to use the cells for subsequent other (protein based) assays position fluorescent-based assays as an efficient alternative. Especially when targeting mutations in postmitotic cells, for example, photoreceptor cells, reporters can help to fine tune therapeutic prime editing, before moving to specialized 2D/3D cell culture or *in vivo* models [19,20].

## Conclusion

Meaningful large animal models such as the pig enter the spotlight of research due to the similar eye size, retinal architecture, and physiology. The presented work shows the quantification of frameshift correction using the prime editing approach causing the deletion of a mutagenic, human homologous adenine insertion within the porcine *ABCA4* exon 43. We generated in total 27 pegRNAs and 4 ngRNAs complexing with the prime editor and measured the frameshift correction rate with a previously described cellular BRET reporter system. Prime editors 2, which requires only pegRNAs, did not result in a significant frameshift correction rate. In combination with additional ngRNAs, nicking the targeted strand leads to highly significant rates of up to 92% in dependence of the distance to the pegRNA-mediated target nicking. Our findings suggest that the BRET reporter system has the potential to easily select potent prime editor/pegRNA/ngRNA combinations for further *in vivo* studies in pigs.

## Supplementary Material

Supplemental data

Supplemental data

Supplemental data

Supplemental data

Supplemental data

Supplemental data

Supplemental data
