# Supplementary material for: Rapid and Reliable Quantification of Prime Editing Targeting Within the Porcine ABCA4 Gene Using a BRET-Based Sensor
Source: Nucleic Acid Ther. 2023 Jun 2;33(3):226–32. doi: 10.1089/nat.2022.0037 (PMC10278032; doi:10.1089/nat.2022.0037)

**Supplementary table 2:** **Table 2.1:** Oligonucleotides used for wild type *ABCA4* target DNA synthesis and subsequent PCR amplification. **Table 2.2:** Mutagenesis PCR primer for adenine insertion.


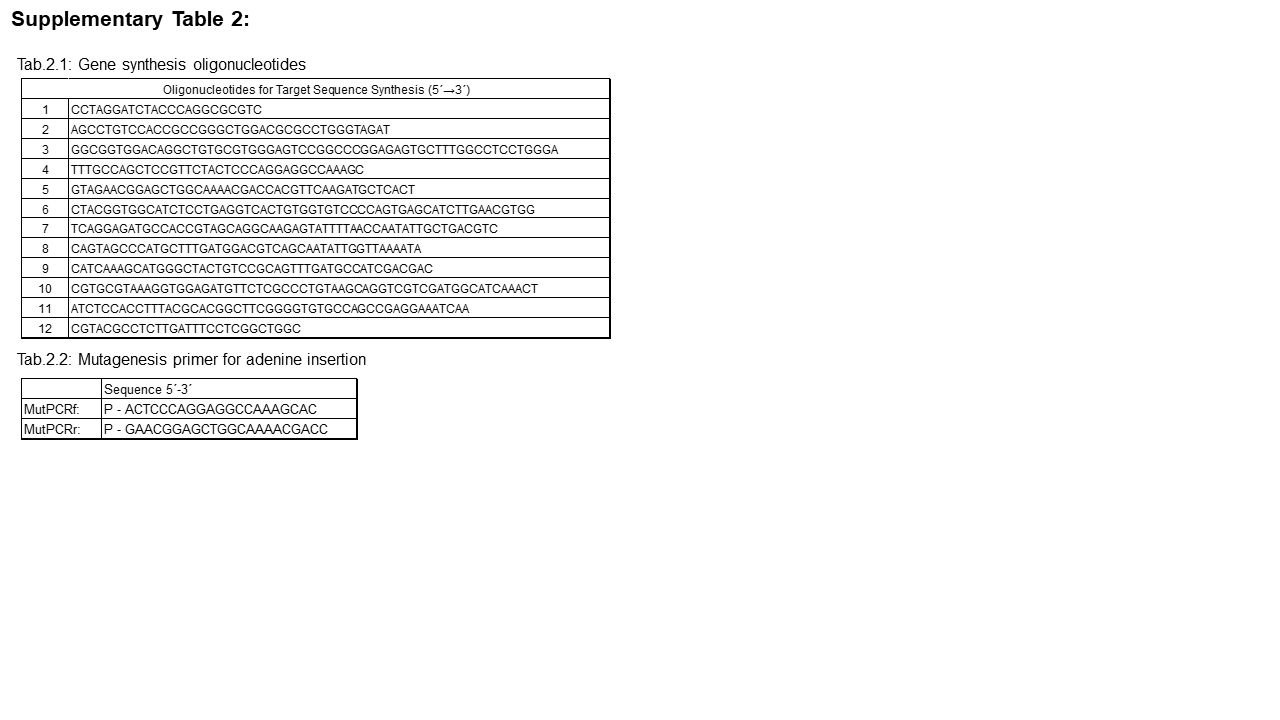

Supplement: Supplemental data [file Suppl_TableS2.docx]
