# Supplementary material for: Rapid and Reliable Quantification of Prime Editing Targeting Within the Porcine ABCA4 Gene Using a BRET-Based Sensor
Source: Nucleic Acid Ther. 2023 Jun 2;33(3):226–32. doi: 10.1089/nat.2022.0037 (PMC10278032; doi:10.1089/nat.2022.0037)

**Supplementary figure 1:** Porcine wild type target sequence synthesis using an oligonucleotide pool with subsequent PCR amplification and in frame integration between the BRET partners RLuc8 and GFP2.


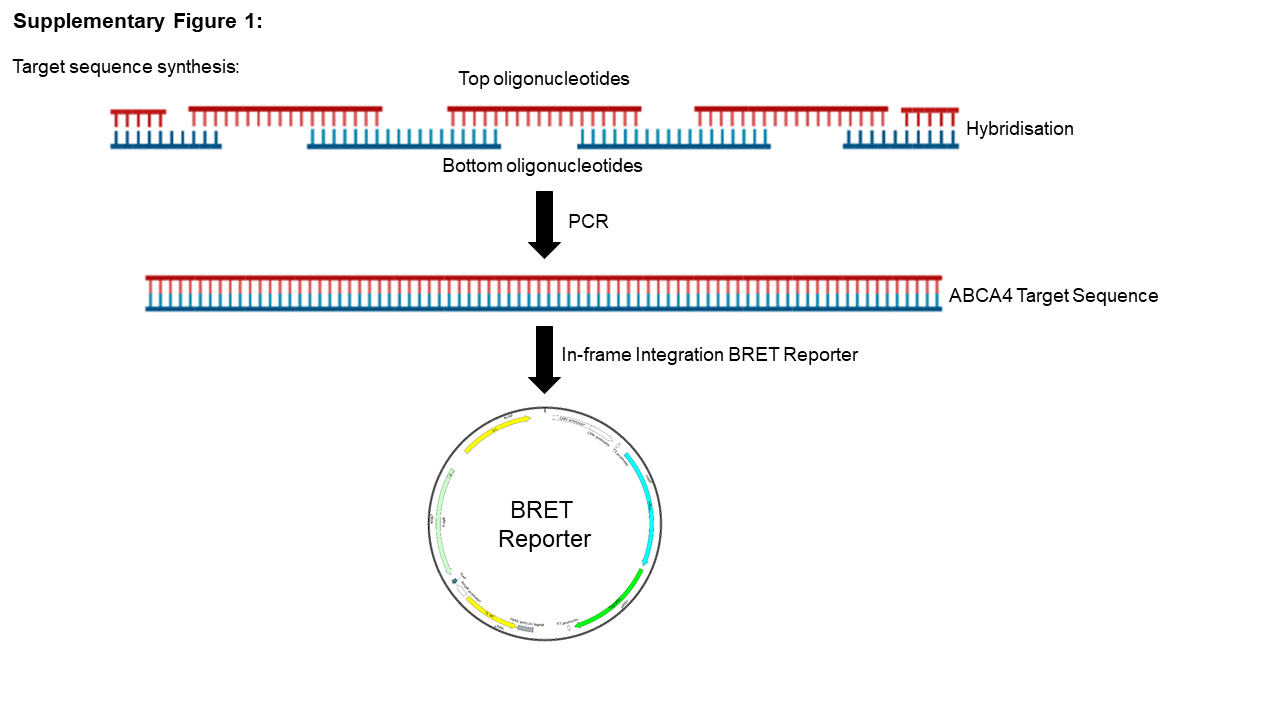

Supplement: Supplemental data [file Suppl_FigureS1.docx]
