# Supplementary material for: Rapid and Reliable Quantification of Prime Editing Targeting Within the Porcine ABCA4 Gene Using a BRET-Based Sensor
Source: Nucleic Acid Ther. 2023 Jun 2;33(3):226–32. doi: 10.1089/nat.2022.0037 (PMC10278032; doi:10.1089/nat.2022.0037)

**Supplementary table 1: Table1.1:** Cloned sequences coding for pegRNAs containing different gRNA portions, a constant scaffold, and varying RTT and PBS length under the control of U6 promotor. **Table 1.2:** Sequences coding for nicking guide RNAs.


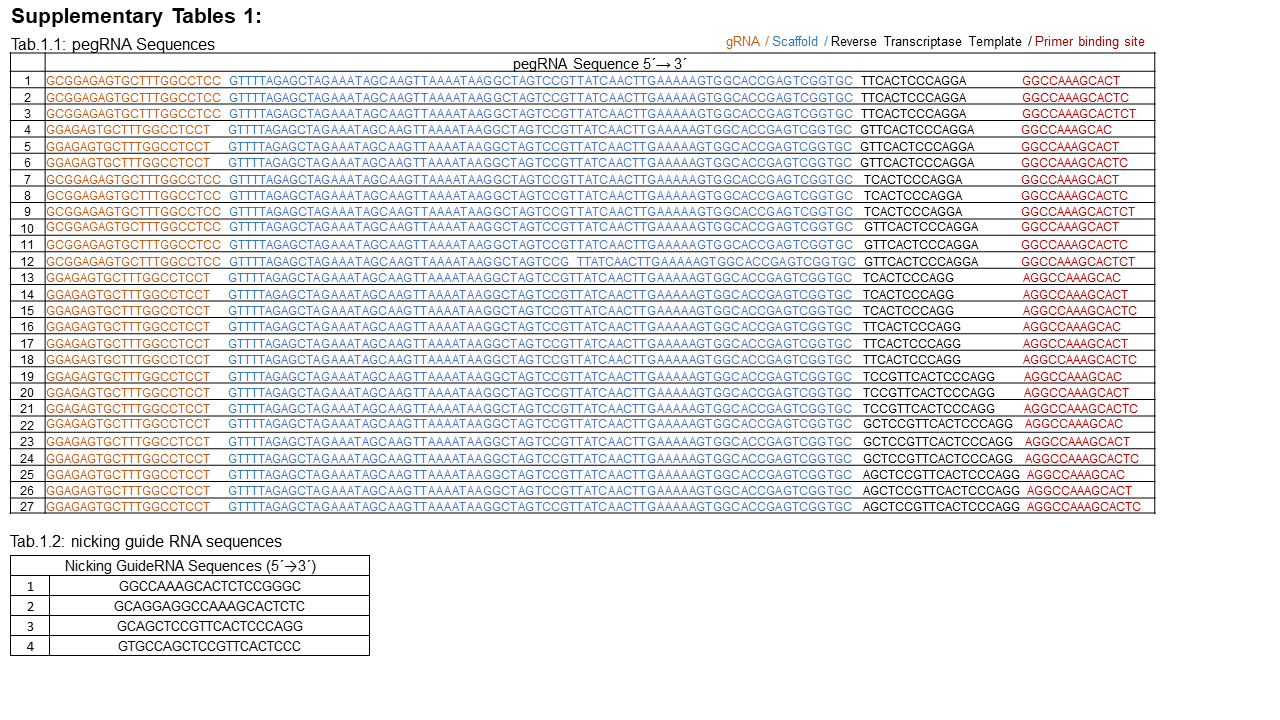

Supplement: Supplemental data [file Suppl_TableS1.docx]
