# Supplementary material for: Rapid and Reliable Quantification of Prime Editing Targeting Within the Porcine ABCA4 Gene Using a BRET-Based Sensor
Source: Nucleic Acid Ther. 2023 Jun 2;33(3):226–32. doi: 10.1089/nat.2022.0037 (PMC10278032; doi:10.1089/nat.2022.0037)

**Supplementary figure 2:** BRET measurements. (A) Raw BRET data of PE2/pegRNA complexes compared to RLuc8 BRET ratio as 0% editing efficiency. (B) Normalized PE2 BRET ratios. (*p<0.05; ***p<0.001)


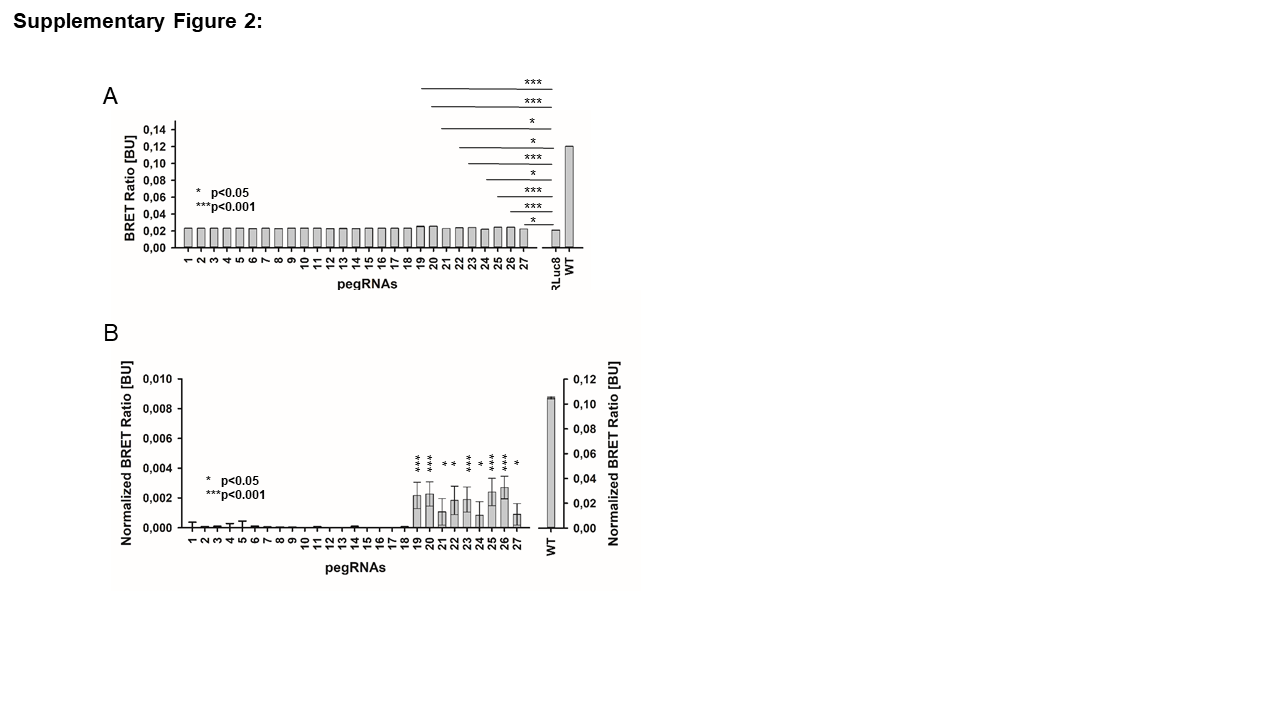

Supplement: Supplemental data [file Suppl_FigureS2.docx]
