# Supplementary material for: Rapid and Reliable Quantification of Prime Editing Targeting Within the Porcine ABCA4 Gene Using a BRET-Based Sensor
Source: Nucleic Acid Ther. 2023 Jun 2;33(3):226–32. doi: 10.1089/nat.2022.0037 (PMC10278032; doi:10.1089/nat.2022.0037)

**Supplementary figure 3:** Fluorescence microscopy of GFP2 reading frame correction after PE2, PE3 and PE3b transfection of selected Prime Editor/pegRNA complexes ± ngRNA 1-4 in HEK293-T cells.


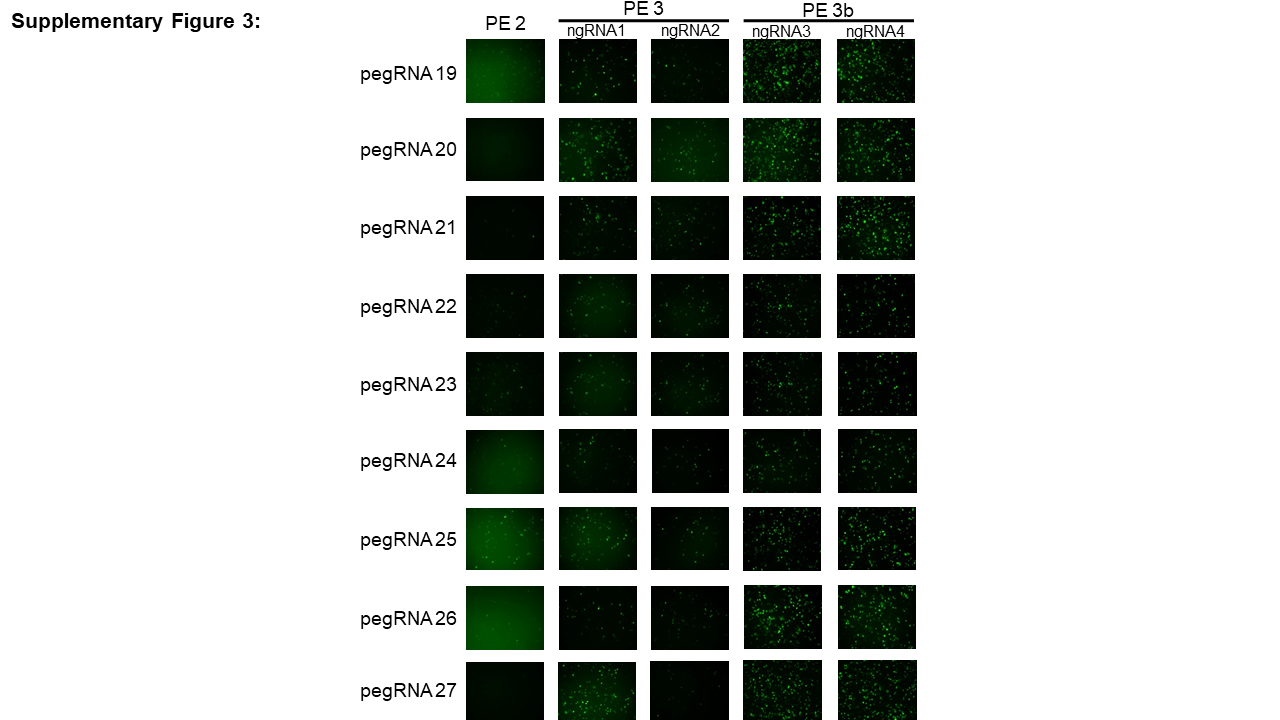

Supplement: Supplemental data [file Suppl_FigureS3.docx]
