# Supplementary material for: Rapid and Reliable Quantification of Prime Editing Targeting Within the Porcine ABCA4 Gene Using a BRET-Based Sensor
Source: Nucleic Acid Ther. 2023 Jun 2;33(3):226–32. doi: 10.1089/nat.2022.0037 (PMC10278032; doi:10.1089/nat.2022.0037)

**Supplementary figure 4:** Statistical analysis of frameshift correction rate [%] using Prime Editors PE2, PE3 and PE3b with pegRNAs 19-27 and additional nicking with ngRNAs 1-4. (***p<0.001)


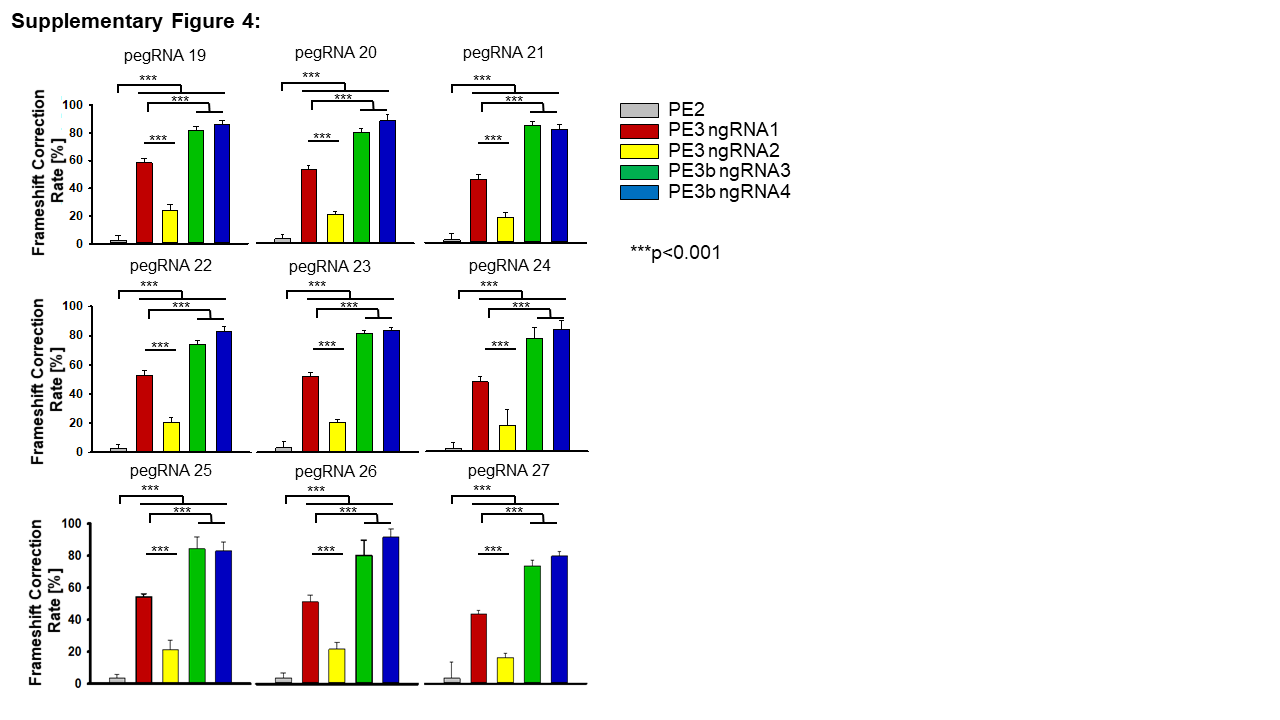

Supplement: Supplemental data [file Suppl_FigureS4.docx]
