# Supplementary material for: Rapid and Reliable Quantification of Prime Editing Targeting Within the Porcine ABCA4 Gene Using a BRET-Based Sensor
Source: Nucleic Acid Ther. 2023 Jun 2;33(3):226–32. doi: 10.1089/nat.2022.0037 (PMC10278032; doi:10.1089/nat.2022.0037)

**Supplementary figure 5:** Western blot confirmation of GFP2 reading frame restoration after PE3b/pegRNA26/ngRNA4 complex application on mutated *ABCA4* in HEK293-T cells.

Supp.Fig.5: Western Blot analysis of Prime Editing. Restoration of *ABCA4* BRET reporter reading frame mediated by PE2 &PE3b.


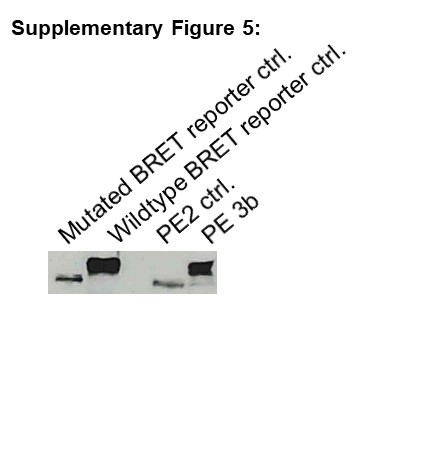

Supplement: Supplemental data [file Suppl_FigureS5.docx]
